# Carbapenemase Detection Methods in Clinical Microbiology: Conventional and Automated Approaches

**DOI:** 10.7759/cureus.112242

**Published:** 2026-07-07

**Authors:** Sonam L Dhavale, Harsha V Patil, Satish R Patil

**Affiliations:** 1 Department of Medical Microbiology, Krishna Institute of Medical Science, Krishna Vishwa Vidyapeeth (Deemed to be University), Karad, IND

**Keywords:** automated systems, carbapenemase, carbapenem-resistant enterobacteriaceae (cre), conventional method, gram-negative bacteria

## Abstract

Due largely to carbapenemase-producing bacteria such as *Pseudomonas aeruginosa*, *Klebsiella pneumoniae*, and *Acinetobacter baumannii*, antimicrobial resistance (AMR) has emerged as a major global health concern. Carbapenems were traditionally thought of as last-line treatments for multidrug-resistant (MDR) Gram-negative infections; however, these pathogens have reduced their efficacy. These pathogens are commonly associated with healthcare-associated infections, which increase morbidity, mortality, and lengthen hospital stays. Therefore, identifying carbapenemase activity is necessary for directing the right course of treatment, implementing infection control strategies, and assisting with antimicrobial stewardship initiatives.

Traditional phenotypic techniques are still widely employed; however, methods such as disk diffusion, minimum inhibitory concentration (MIC) testing, and the modified Hodge test (MHT) are constrained by their slow turnaround times and challenges in identifying particular resistance mechanisms. Although enhanced sensitivity and specificity are provided by sophisticated biochemical assays such as Carba NP, Blue-Carba, and ethylenediaminetetraacetic acid (EDTA)-based techniques, problems with false positives and emerging variants still exist. Although cost and infrastructure requirements frequently limit their adoption, automated systems such as VITEK 2, BD Phoenix, MicroScan WalkAway, and matrix-assisted laser desorption ionization-time of flight mass spectrometry (MALDI-TOF MS) improve diagnostic accuracy through quick, consistent, and repeatable results. Future developments, including AI-driven diagnostics, rapid point-of-care tools, genomic surveillance, and portable devices, highlight the urgent need for accessible solutions to improve patient outcomes, boost diagnostic capacity, and stop the spread of carbapenem resistance worldwide.

## Introduction and background

Antimicrobial resistance (AMR) develops when microorganisms survive or multiply despite exposure to antimicrobial agents intended to eliminate them [1]. It poses a major global health challenge, threatening the prevention and treatment of infections caused by bacteria, viruses, parasites, and fungi, and has been acknowledged as a significant public health concern since the early 2010s [1]. Carbapenemases are enzymes capable of hydrolyzing carbapenems, monobactams, cephalosporins, and penicillins. Carbapenems are often the preferred treatment for multidrug-resistant (MDR) Gram-negative infections; however, their extensive use has resulted in increasing cases of carbapenem-resistant Enterobacteriaceae (CRE), carbapenem-resistant *Pseudomonas aeruginosa* (CRPA), and carbapenem-resistant *Acinetobacter baumannii* (CRAB). Accurate and timely detection of carbapenem-resistant strains is essential for patient management and preventing community spread [2]. The majority of clinical microbiology laboratories depend on antimicrobial susceptibility testing (AST) without investigating the mechanisms of carbapenem resistance. While AST guides antibiotic selection, identifying carbapenemase production and its type is crucial, especially when susceptibility data for newer agents are unavailable [3].

Carbapenemases vary in their ability to hydrolyze β-lactams. Their detection is critical for infection control, as they are associated with widespread resistance, treatment failure, and increased mortality. Transmissible metallo-β-lactamases (MBLs) are particularly concerning because they inactivate nearly all β-lactams, including carbapenems. These enzymes can be unpredictably acquired by nosocomial pathogens such as *P. aeruginosa*, *A. baumannii*, and Enterobacteriaceae, whereas chromosomal carbapenemases are typically found in less common pathogens like *Stenotrophomonas maltophilia*, Aeromonas spp., and Chryseobacterium spp. [2]. Conventional AST, though reliable, requires two to five days, limiting its use in urgent cases. Rapid molecular assays, such as Carba-R GeneXpert, provide results within two to three hours. Phenotypic methods, including Carba M, Modified Carba NP, modified carbapenem inactivation method (mCIM), and ethylenediaminetetraacetic acid (EDTA)-carbapenem inactivation method (eCIM), are also useful for identifying carbapenemase activity. Gram-negative organisms such as *Klebsiella pneumoniae*, *P. aeruginosa*, and *A. baumannii* frequently exhibit carbapenem resistance, either through intrinsic mechanisms or via transferable carbapenemase genes [4].

This review aims to summarize current methods for carbapenemase detection and evaluate their strengths and limitations. To achieve this, relevant literature indexed in PubMed, Scopus, and Google Scholar has been systematically analyzed [5]. The CDC, or Centers for Disease Control and Prevention, defines CRE as Enterobacteriaceae able to withstand at least one carbapenem (e.g., imipenem (IPM), ertapenem (ETP), doripenem, or meropenem (MEM)) or those producing carbapenemase enzymes that confer resistance [6].

## Review

Research strategy

**Figure 1:**
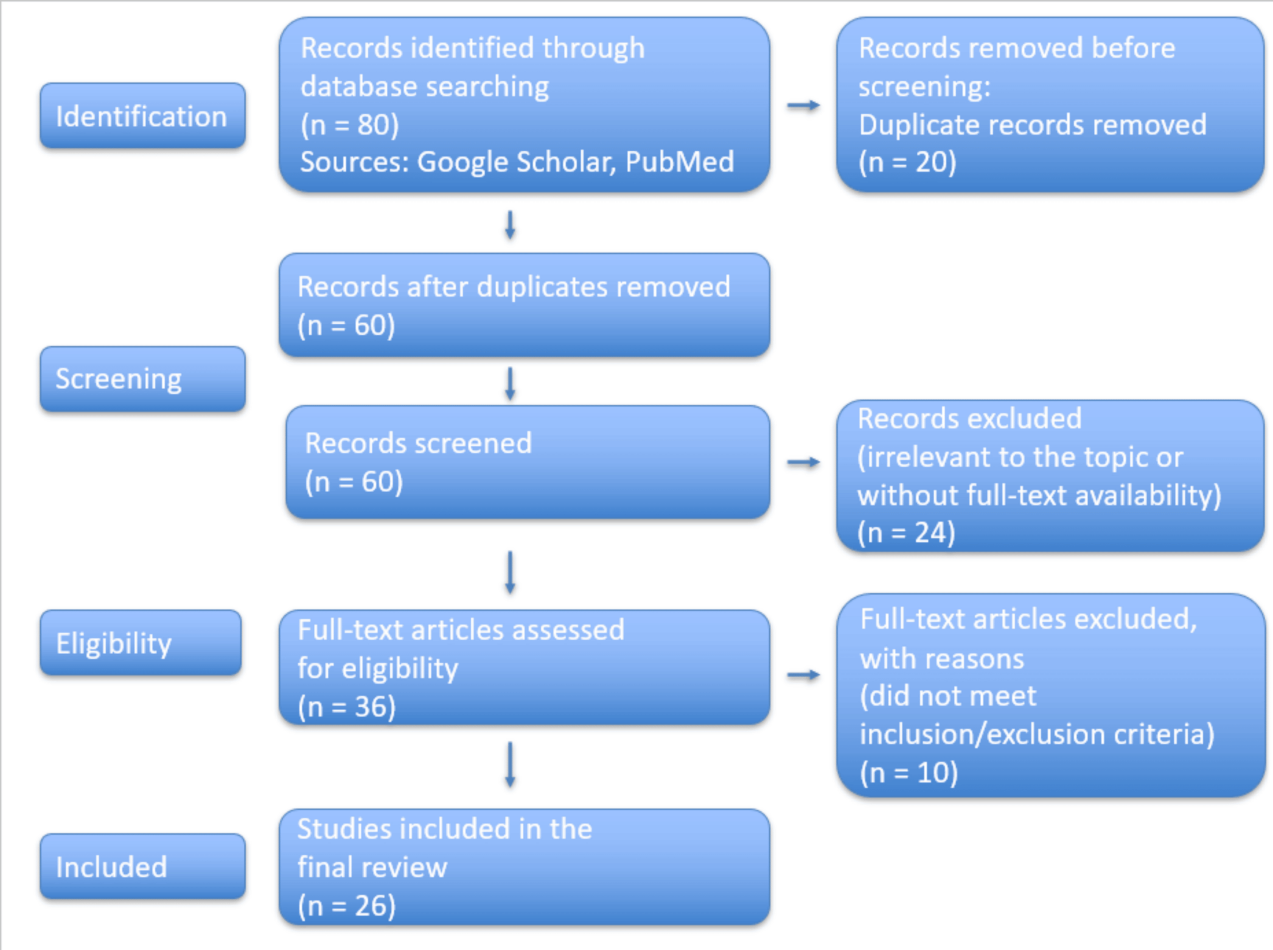
Article selection and screening process Image credit: Created by authors using Microsoft PowerPoint (Microsoft Corporation, Redmond, WA, USA).

A thorough literature search was conducted using databases such as Google Scholar and PubMed to develop this review on carbapenemase detection methods in clinical microbiology. MeSH terms and keywords included “conventional methods,” “carbapenem resistant enterobacteriaceae (cre),” “automated systems,” “gram negative bacteria,” and “carbapenemase,” combined using Boolean operators (AND, OR). Articles published in English from 2014 to 2025 were included (Figure 1). Inclusion criteria comprised original research articles, reviews, and surveillance studies focusing on carbapenemase-producing *Escherichia coli*. Studies not available in English, lacking full text, or not directly relevant to the topic were excluded. Titles and abstracts were screened initially, followed by full-text evaluation. Reference lists of included studies were also manually searched to identify additional relevant publications. The study selection process is presented using a PRISMA flow diagram.

Overview of carbapenemases

Gram-negative microbes that have become resistant to carbapenems, which are thought of as last-resort treatments for serious infections, are known as carbapenem-resistant organisms (CROs). Carbapenemase production, which includes Class A serine β-lactamases (*Klebsiella pneumoniae* carbapenemase (KPC), Guiana extended-spectrum β-lactamase (GES), imipenem-hydrolyzing β-lactamase (IMI)), Class B MBLs (New Delhi metallo-β-lactamase (NDM), Verona integron-encoded metallo-β-lactamase (VIM), imipenemase (metallo-β-lactamase) (IMP)), and Class D oxacillinases (OXA; OXA-23, OXA-48, OXA-58), is the main mechanism. Non-carbapenemase mechanisms, including porin loss, overexpression of efflux pumps, and hyperproduction of extended-spectrum β-lactamases (ESBLs) or AmpC enzymes, further contribute to resistance. These pathogens are often extensively drug-resistant (XDR) or MDR, which significantly limits treatment options and increases mortality, especially in hospital outbreaks. Interspecies and regional distribution are made easier by their quick spread, which is mostly caused by plasmid-driven horizontal gene transfer. Clinically, CROs are linked to wound, urinary, respiratory, and bloodstream infections, which frequently result in extended hospital stays and treatment failure. Accurate detection using phenotypic assays and molecular platforms is essential to guide treatment, implement infection control strategies, and reduce adverse patient outcomes.

Classification of carbapenemases

Ambler Classification

Class A carbapenemases (KPC): Based on evolutionary studies, carbapenemases can be classified into six different classes. Four of these groups comprise enzymes from the GES, KPC, SME, and IMI/NMC-A families; SHV-38 and SFC-1 each belong to a different group. The genes encoding these carbapenemases can be found on plasmids or on the chromosomes of the host organism. While bla(KPC) genes and a single bla(IMI-2) gene are surrounded by transposable elements on plasmids, bla(GES) genes are usually located within gene cassettes on class I integrons. Penicillins, cephalosporins, monobactams, IPM, and MEM are among the drugs that Class A carbapenemases can hydrolyze. The Bush-Jacoby-Medeiros classification system further separates these enzymes into four phenotypically diverse groups: 2br, 2be, 2e, and 2f. Like other Class A β-lactamases, Class A carbapenemases can be inhibited by β-lactamase inhibitors such as clavulanate and tazobactam [7].

Class B carbapenemases (NDM, VIM, IMP): A useful summary and update on epidemiology has been provided by a recent thorough analysis of metallo-lactamases. This specific class of lactamases is distinguished by its capacity to hydrolyze carbapenems, resistance to widely used lactamase inhibitors, and susceptibility to inactivation by metal ion chelators. Aztreonam cannot be hydrolyzed by these enzymes, despite their wide variety of substrates, which include cephalosporins and penicillins. Lactams are particularly vulnerable to inhibition by EDTA, a chelator of Zn2+ and other divalent cations, because the hydrolysis mechanism depends on the interaction between lactams and zinc ions in the enzyme's active site. Chromosomal enzymes found in environmental and opportunistic pathogenic bacteria such as *Bacillus cereus*, Aeromonas spp., and *S. maltophilia* were the first metallo-lactamases to be discovered and studied. These lactamases were inducible after exposure to lactams, and these particular enzymes were commonly found in bacteria that also produced at least one serine lactamase. Fortunately, these bacteria, aside from *S. maltophilia*, are not frequently linked to serious nosocomial infections because they are typically opportunistic pathogens, and their chromosomal metallo-lactamase genes are difficult to transmit. Early classification based on functional investigations of isolated proteins distinguished these enzymes from other classes that used a serine-based hydrolytic mechanism. The requirement for Zn2+ for effective lactam hydrolysis and resistance to inhibition by clavulanic acid and tazobactam were notable distinctions. Their capacity to hydrolyze carbapenems was a distinguishing feature of their substrate spectrum [8].

Class D carbapenemases (OXA-48): At first, class D β-lactamases, also called OXA, were uncommon and spread by plasmids. Compared to conventional penicillins, they prefer to break down oxazolyl penicillins such as cloxacillin, methicillin, and oxacillin more quickly. They do not, however, work as well against first-generation cephalosporins. The term "OXA" comes from oxacillin, which is their preferred substrate. While the rest of the molecule has different amino acid sequences, the active region of these enzymes has a conserved serine-based structure. Metal chelators and β-lactamase inhibitors do not affect them. These enzymes appeared at the same time that methicillin and flucloxacillin were initially employed to treat staphylococcal infections. Early OXA β-lactamases, such as OXA-1, OXA-2, and OXA-3, were found in Gram-negative bacteria and mainly served as penicillinases, hydrolyzing oxacillin more effectively than penicillin. The first extended-spectrum OXA variation, OXA-11, was then found in *P. aeruginosa* and showed improved ceftazidime hydrolysis. Despite the fact that they were not readily distributed, *P. aeruginosa* also contained other extended-spectrum OXA enzymes, including OXA-13, OXA-14, OXA-15, OXA-16, OXA-17, OXA-19, OXA-28, and OXA-45. Currently, groups such as OXA-23-like, OXA-24/40-like, OXA-48-like, OXA-58-like, OXA-143-like, and OXA-235 are among the OXA enzymes with carbapenem-hydrolyzing activity [2].

**Figure 2:**
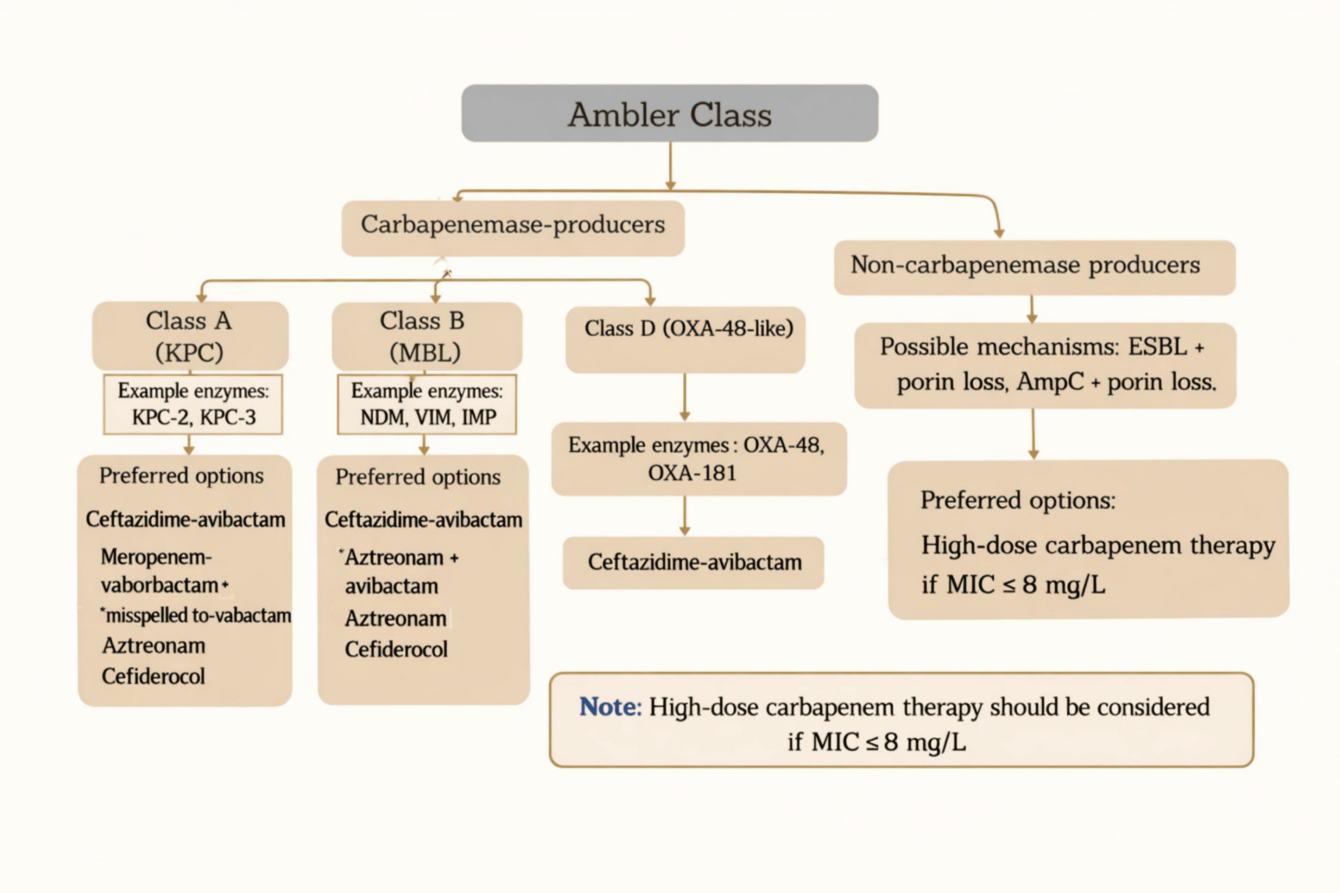
Classification of carbapenemase Classification of carbapenem resistance mechanisms according to the Ambler classification. CRE are divided into carbapenemase-producing and non-carbapenemase-producing isolates. Carbapenemase producers include Class A serine carbapenemases (KPC), Class B MBLs (NDM, VIM, IMP), and Class D OXA (OXA-48-like enzymes). Non-carbapenemase-mediated resistance may result from ESBL or AmpC β-lactamase production combined with porin loss. The figure also highlights representative enzymes and preferred therapeutic options for each resistance mechanism. CRE: carbapenem-resistant Enterobacteriaceae; MBLs: metallo-β-lactamases; ESBL: extended-spectrum β-lactamase; MIC: minimum inhibitory concentration; ESBL: extended-spectrum β-lactamase; KPC: *Klebsiell pneumoniae* carbapenemase; MBL: metallo-β-lactamase; MDM: New Delhi metallo-β-lactamase; VIM: Verona integron-encoded metallo-β-lactamase; IMP: imipenemase (metallo-β-lactamase); OXA: oxacillinase Image credit: Created by authors using Canva (Canva Pty Ltd., Sydney, NSW, Australia).

Classification of carbapenem resistance mechanisms according to the Ambler classification (Figure 2).

Detection and characterization of carbapenemase resistance

**Figure 3:**
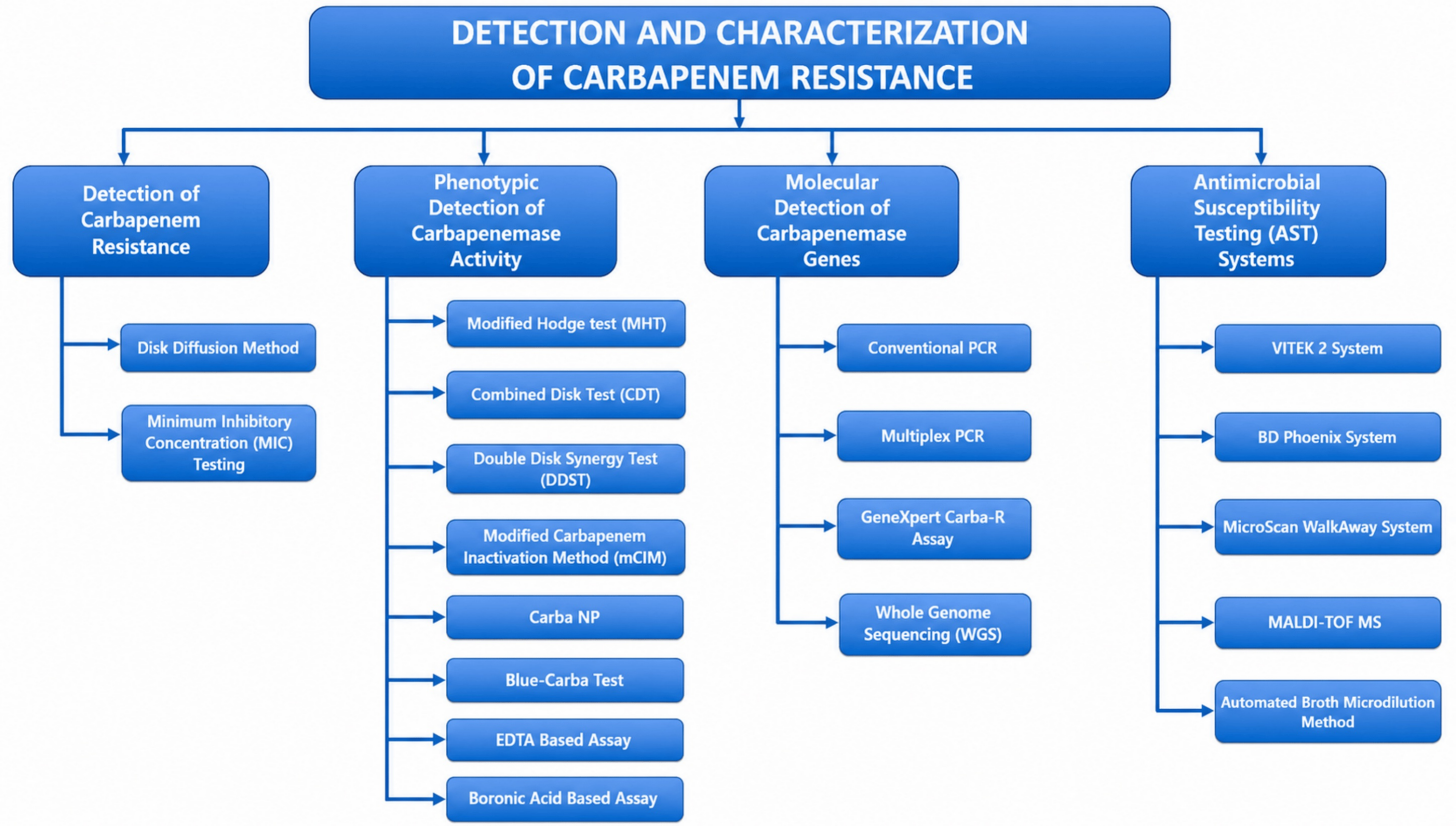
Detection and characterization of carbapenemase resistance MALDI_TOF MS: matrix-assisted laser desorption ionization-time of flight mass spectrometry, BD: Becton Dickinson; EDTA: ethylenediaminetetraacetic acid; PCR: polymerase chain reaction Image credit: Created by authors using Microsoft PowerPoint (Microsoft Corporation, Redmond, WA, USA).

CROs can be identified using a variety of diagnostic approaches. These methods can be broadly categorized into the detection of carbapenem resistance, phenotypic detection of carbapenemase activity, molecular identification of carbapenemase genes, and AST systems (Figure 3).

Detection of Carbapenem Resistance

Disk diffusion method: Disk diffusion testing was performed using standardized antibiotic-impregnated discs on Mueller-Hinton agar (MHA) plates inoculated with the examination organism. The organism's susceptibility was reflected in the zone of inhibition that resulted from the antibiotic diffusing into the medium during incubation. This zone's diameter was directly correlated with the bacterium's sensitivity. Since it might be difficult to differentiate between intrinsic and acquired resistance, precise bacterial species identification is still crucial even if disc diffusion is a very reliable method for regular susceptibility testing. The Clinical and Laboratory Standards Institute (CLSI) disk diffusion assay was employed to measure antimicrobial susceptibility, and CLSI standards were used to establish interpretive breakpoints. Zones of inhibition ≥23 mm were classified as susceptible, 20 mm-22 mm as intermediate, and ≤19 mm as resistant for the carbapenems IPM, ETP, and MEM [9].

Minimum inhibitory concentration: The minimum inhibitory concentration (MIC) is considered the gold-standard quantitative method in clinical microbiology for determining the lowest concentration of an antibacterial agent that totally prevents visible bacterial growth [10]. In this assay, a standardized bacterial inoculum is exposed to two-fold serial dilutions of an antibiotic, typically ranging from 0.5 µg/mL to 16 µg/mL, using broth microdilution or agar dilution techniques. A fresh bacterial colony suspension is adjusted to a 0.5 McFarland standard and further diluted in cation-adjusted Mueller-Hinton broth (CAMHB) to achieve a final inoculum of approximately 5×10⁵ CFU/mL per well in a 96-well microtiter plate. The plates are then covered or inverted and incubated at 35±2°C for 16-20 hours, or up to 24 hours for certain resistant isolates. Wells showing turbidity, a button, or a pellet at the bottom indicate bacterial growth, signifying that the antibiotic concentration was insufficient to inhibit multiplication [10,11]. The MIC endpoint is defined as the lowest concentration in the dilution series that demonstrates no visible growth, representing the precise quantitative limit of inhibition [12]. This value is subsequently compared with established interpretive breakpoints to classify the isolate as susceptible, intermediate, or resistant [11].

Phenotypic Detection of Carbapenemase Activity

Modified Hodge test: American Type Culture Collection (ATCC) 25922 *E. coli* was employed as the indicator strain in the modified Hodge test (MHT) for the detection of carbapenemase production. An *E. coli* ATCC 25922 suspension was prepared in broth or saline, adjusted to a 0.5 McFarland standard, and subsequently diluted 1:10. A 10 µg ETP or MEM disc was placed at the center of a lawn culture prepared on MHA using the diluted suspension. Using a sterile swab, two to five colonies of the organism under test were streaked in a straight line, approximately 20-25 mm in length, from the edge of the carbapenem disc toward the periphery of the plate. The plates were kept in an incubator at 37 °C for 16-24 hours. Following incubation, the existence of a cloverleaf-like indentation at the intersection between the indicator strain and the test organism was interpreted as a positive result, confirming carbapenemase activity [13].

Combined disk test: The combined disk test (CDT), also referred to as the disk potentiation test, is a simple yet essential phenotypic confirmation method widely used in clinical microbiology laboratories to identify specific resistance mechanisms. It is particularly valuable for differentiating carbapenemase classes such as KPC and MBL in Gram-negative bacilli [14]. The principle of the assay involves comparing the inhibition zone of a standard β-lactam antibiotic disk with that of a disk containing the same antibiotic in combination with a specific enzyme inhibitor. A fresh bacterial colony suspension, adjusted to a 0.5 McFarland standard, is lawn-cultured uniformly on MHA using a sterile swab [14,15]. For carbapenemase detection, a MEM disk (10 µg) is positioned next to carbapenem disks supplemented with inhibitors such as EDTA for Class B MBLs [14,16]. The plates are inverted and incubated at 35±2°C for 16-24 hours. A positive result is indicated when the inhibition zone diameter around the inhibitor-supplemented disk is ≥5 mm larger than that of the antibiotic disk alone. Conversely, if the zone difference is <5 mm, the inhibitor has no effect, suggesting that resistance is mediated by an alternative mechanism [14,17].

Double disk synergy test: The double disk synergy test (DDST) is a traditional and reliable phenotypic confirmatory method widely employed in clinical microbiology laboratories to detect ESBL production in Gram-negative bacteria such as *K. pneumoniae* and *E. coli* [14,18]. The principle of the assay is based on assessing the spatial synergy between a β-lactamase inhibitor, typically clavulanic acid, and an extended-spectrum oxyimino-cephalosporin [18,19]. A fresh bacterial suspension adjusted to a 0.5 McFarland standard is uniformly lawn-cultured on MHA using a sterile swab [14,20]. Target cephalosporin disks such as cefotaxime (CTX, 30 µg) or ceftazidime (CAZ, 30 µg) are placed at a critical distance of 20-30 mm (center-to-center) from an amoxicillin-clavulanic acid (AMC, 20/10 µg) disk positioned at the center of the plate [21]. The plates are inverted and incubated for 16-24 hours. A positive result is indicated by an asymmetrical expansion or bulging of the cephalosporin inhibition zone toward the AMC disk, producing a characteristic “keyhole” or “champagne-cork” appearance. This phenomenon occurs because clavulanic acid diffuses outward and neutralizes ESBL enzymes, thereby restoring cephalosporin activity [19,20]. In contrast, a negative result is characterized by symmetrical, circular inhibition zones around the cephalosporin disks, signifying the absence of enzyme-inhibitor synergy and thus no ESBL production [19,20].

Modified carbapenem inactivation method: The method of modified mCIM is a reliable phenotypic screening assay frequently employed in clinical microbiology laboratories to identify the production of carbapenemase in Gram-negative bacilli, including Enterobacteriaceae and *P. aeruginosa* [21,22]. In this method, a standard inoculum of the test organism (1-µL loopful for Enterobacteriaceae or a 10-µL loopful for *P. aeruginosa*) is emulsified in Tryptic soy broth (TSB; 2 mL) containing a 10-µg MEM disc and incubated at 35°C for four hours. If the strain produces carbapenemase enzymes, the antibiotic within the disc is hydrolyzed and inactivated [23,24]. Following incubation, the disc is removed and placed onto an MHA plate previously inoculated with a lawn of carbapenem-susceptible *E. coli* ATCC 25922, which serves as the indicator strain. The plate is further incubated for 18-24 hours. Following that, the inhibitory zone around the disc is measured. A zone diameter of 6-15 mm, or 16-18 mm with pinpoint satellite colonies, indicates a positive result, confirming carbapenemase activity [25,26]. Conversely, a clear inhibition zone of ≥19 mm signifies a negative result, demonstrating that the MEM remained intact and effectively prevented the growth of the indicator strain [24].

Carba NP: A quick colorimetric microtube technique called the Carba NP assay uses IPM hydrolysis to identify carbapenemase activity. Acidic byproducts produced by hydrolytic activity can be seen as a clear color shift in a pH indicator. In 100 µL of 20 mM Tris-HCl lysis buffer, a loopful of bacterial growth from MHA is suspended and quickly vortexed. This suspension is then combined with 100 µL of phenol red indicator solution that has been pre-adjusted to pH 7.8 and contains 0.1 mmol/L ZnSO4. IPM powder (3 mg/0.5 mL) is added to the reaction tube, whereas the control tube contains only the antibiotic-free indicator solution. Both tubes are vortexed for roughly ten seconds. The solutions appear red to orange before incubation. After incubation at 37°C for up to two hours, isolates that generate carbapenemase exhibit a color shift from reddish-orange to pale orange, dark yellow, or yellow, whereas those that do not maintain their original color. This simple test offers a trustworthy phenotypic confirmation of the formation of carbapenemase [25].

Blue-Carba test: The Carba NP test has been simplified and made more affordable with the launch of the Blue-Carba assay by Pires et al. (2013). Using complete bacterial cells, a less expensive source of IPM, and bromothymol blue as the pH indicator, this method eliminates the protein extraction phase. The indicator has an ideal detection range (pH 6.0-7.6) for the majority of β-lactamases, and it quickly changes color within two hours, increasing speed and cost-effectiveness. Published studies report sensitivity and specificity generally ranging from 90% to 100%, depending on the carbapenemase type and bacterial species; however, greater inoculum doses are needed to detect OXA-48 enzymes. Despite its encouraging performance, there is still a lack of validation, and more research is necessary to ascertain its dependability across all class D carbapenemases [26].

Ethylenediaminetetraacetic acid-based assays: CLSI 2023 has standardized the EDTA-based tests, mCIM and eCIM, for the identification and distinction of Enterobacterales that produce carbapenemases. While the eCIM uses EDTA, a zinc-chelating agent, to specifically inhibit MBLs, the mCIM assesses carbapenemase activity by incubating MEM disks with bacterial suspensions. Two tubes of Trypticase Soy Broth are prepared for each isolate; one is supplemented with EDTA, and the other is used as a control. Following MEM disk inoculation and incubation, the disks are placed on MHA seeded with *E. coli* ATCC 25922. Inhibition zones of 6-15 mm or 16-18 mm with colonies present within the zone indicate a positive mCIM result. Only when mCIM is positive can eCIM be interpreted; an increase of at least 5 mm in the zone of inhibition relative to mCIM strongly suggests the presence of MBL production. When combined, these tests offer a reliable phenotypic method for verifying carbapenemase activity and differentiating between MBLs and serine carbapenemases, which enhances diagnostic accuracy in clinical microbiology laboratories [3].

Boronic acid-based assays: A phenotypic method for class identification is the boronic acid-based assay. KPC is one type of carbapenemase. In particular, these enzymes are inhibited by phenyl boronic acid, which increases the inhibitory action when paired with β-lactam antibiotics, making detection easier. Twenty micrograms of phenyl boronic acid is dissolved in 1 mL of dimethyl sulfoxide to create a stock solution, from which 20 µL, or 400 µg of boronic acid, is used as the working solution. The positive control strain is *K. pneumoniae* BAA-1705, while the negative control strain is *K. pneumoniae* BAA-1706. The test isolate is prepared as a 0.5 McFarland suspension, streaked as a lawn on MHA, and antibiotic disks (IPM, MEM, ETP, cefepime, cefoxitin, cefotetan, cefotaxime, and ceftazidime) with and without boronic acid are applied. After overnight incubation at 37°C, the inhibition zones are compared to interpret the results. KPC production is thought to be indicated by an increase of at least 5 mm in the zone diameter surrounding the boronic acid-containing disk when compared to the corresponding antibiotic disk alone. This assay offers a simple and trustworthy way to identify Class A carbapenemases and differentiate between KPC producers and non-producers in standard clinical microbiology practice [2].

Methodological Strengths and Clinical Utility of Conventional Phenotypic Assays

Due to their accessibility, cost, and ease of use, traditional carbapenemase detection techniques are still useful. While MIC testing yields exact quantitative resistance levels, disk diffusion is standardized and reasonably priced. Inhibitor-based assays like CDT and DDST allow for the visual distinction of carbapenemase types, whereas the MHT provides a simple laboratory setup for KPC detection. Selective chromogenic media provide quick screening straight from patient samples, while more advanced techniques like CIM/mCIM provide high sensitivity and specificity with strong concordance to PCR. When combined, these methods provide useful, affordable tools that facilitate routine diagnostics, especially in environments with limited resources.

Technical Vulnerabilities and Diagnostic Challenges of Phenotypic Testing

Phenotypic assays such as disk diffusion, MIC testing, MHT, CDT, DDST, Carba NP, and inhibitor-based methods (EDTA, boronic acid) have some disadvantages, despite the fact that they are still vital instruments in typical microbiology laboratories. Technical flaws include false-positive and false-negative results, particularly in assays such as MHT and EDTA-based synergy testing, where non-specific interactions may obscure real carbapenemase activity. Sensitivity is often reduced for novel or uncommon variants, as shown by OXA-48 detection in Blue-Carba tests, and differences in inoculum size, incubation conditions, and medium composition further compromise reproducibility. Diagnostic issues arise from the time-consuming nature of confirmatory tests such as mCIM, which require prolonged incubation and delay treatment decisions. Many laboratories are unable to use cutting-edge techniques because of inadequate resources, requiring the use of traditional disk diffusion or MIC techniques, which might not be able to distinguish between intrinsic and acquired resistance. Furthermore, variable interpretations of inhibition zones and synergy patterns result from the absence of established techniques among institutions, which compromises comparability. The use of automated or chromogenic systems is further hampered by infrastructure and financial constraints, forcing smaller laboratories to rely on manual phenotypic techniques. All of these weaknesses and difficulties point to the necessity of combining molecular confirmation with phenotypic screening to guarantee precise, prompt, and clinically significant identification of carbapenemase-producing organisms.

Molecular Detection of Carbapenemase Genes

Conventional polymerase chain reaction: Real-time PCR, which uses fluorescent probes or dyes to simultaneously detect and quantify resistance genes, is replacing conventional PCR followed by gel electrophoresis. In melting curve analysis, the data are shown on a screen as fluorescence intensity against melting temperature. Without the requirement for gel electrophoresis, genes can be instantly identified using their distinct melting curves. Other real-time assays are highly selective because they employ probes with distinct molecular fluorescent beacons that bind to the template DNA and undergo ligation (probe ligation). This is followed by universal primer amplification and fluorescent detection of the distinct beacons and probes using their cycle threshold, a technique utilized by numerous commercial microarrays, including the Check-Direct CPE [26].

Multiplex polymerase chain reaction: Highly specific primers that do not self-react are necessary for multiplex PCR, which uses a set of several primers to detect multiple genes at once. For simple resolution and identification, the target genes' amplicon sizes must differ, and the cycling conditions must be optimal for each primer. A variety of resistance genes can now be detected in a single reaction with a faster turnaround time by combining real-time and multiplex PCR [26].

GeneXpert Carba-R assay: Real-time PCR cartridges are used by the GeneXpert system (Cepheid, Sunnyvale, CA) to quickly identify clinically significant carbapenemase genes such as blaKPC, blaIMP, blaVIM, blaNDM, and blaOXA-48. However, OXA-48-like variants are difficult to diagnose because PCR is unable to distinguish them; sequencing is necessary for confirmation. While OXA-181 exhibits activity comparable to OXA-48 despite amino acid changes, variants such as OXA-163 confer resistance to broad-spectrum cephalosporins with limited carbapenem hydrolysis. The reported inability of the Xpert Carba-R cartridge to detect blaOXA-48 in *E. coli* highlights the limitations of molecular assays, which may overlook some genes and should not yet be regarded as reference standards for CRE detection. After incubation and DNA extraction, the Check-MDR Carba assay (Check-Points), which is based on multiplex PCR, effectively detects KPC, NDM, OXA-48, VIM, and IMP from rectal and throat swabs; results are available within 5.5 hours. For the majority of carbapenemases, however, the Xpert Carba-R kit provides quicker detection (52 minutes) with excellent sensitivity and specificity; nonetheless, for OXA-48 and OXA-181 variants, specificity decreases to 71%. All things considered, even if these molecular platforms offer quick detection, more testing is necessary to prove their dependability and repeatability as all-encompassing diagnostic instruments [26].

Whole genome sequencing: A sophisticated genotypic technique for the thorough identification and description of carbapenemase-producing Gram-negative bacteria is whole genome sequencing (WGS). By examining the entire genetic content of CROs, next-generation sequencing (NGS) technologies offer a powerful platform for their detection, classification, and characterization. Multiple resistance genes, plasmid replicons, and mobile genomic elements that propel the spread of carbapenemase determinants, including blaNDM, blaKPC, blaVIM, and blaOXA-48, can all be simultaneously identified using WGS. DNA extraction, library preparation, sequencing using platforms such as Oxford Nanopore or Illumina, bioinformatic assembly, and annotation are the usual steps in the approach. Despite its excellent sensitivity and specificity, its constraints include the need for extensive data storage capacity, standardized bioinformatic pipelines for data processing, and significant computational resources. However, WGS is a key component of contemporary molecular microbiology because it offers unmatched resolution for epidemiological surveillance, outbreak investigation, and evolutionary tracking of carbapenemases [26].

Antimicrobial Susceptibility Testing Systems

VITEK 2 system: Currently used in more than a thousand laboratories throughout India, the VITEK 2 system (bioMérieux) is a widely used automated platform for AST and microorganism identification (ID/AST). Its detection method is based on sophisticated colorimetric analysis, which interprets biochemical processes in microwells with lyophilized substrates using the analytical profile index (API) concept. In order to accurately identify clinical isolates, the system quickly compares their biochemical profiles to a large library of reference species using both endpoint and kinetic analyses. Typically, results are available within three to eighteen hours, depending on the type of card and organism. With specific ID cards designed for Gram-positive, Gram-negative, anaerobic, and fastidious bacteria, as well as yeasts, VITEK 2 exhibits excellent performance in terms of accuracy, repeatability, and efficiency. Its uses go beyond standard diagnostics to support hospital surveillance systems, epidemic investigations, and antimicrobial stewardship measures, highlighting its significance as a key technology in contemporary clinical microbiology [27].

BD Phoenix system: Currently used in over 100 laboratories across India, the BD Phoenix system is an automated platform for microbial identification and antimicrobial susceptibility testing (ID/AST). It can perform 50 or 100 tests at once and comes in two configurations: Phoenix M50 and M100. The method uses customized panels, such as combination, AST-only, and ID-only panels, each of which has 51 microwells filled with dried biological substrates. Conventional, chromogenic, and fluorogenic biochemical assays that evaluate microbial metabolism and substrate usage are used to accomplish identification. Chromogenic substrates produce color shifts during enzymatic hydrolysis, fluorogenic substrates release fluorescent derivatives, and changes in the phenol red indicator reflect acid production. A thorough biochemical profile is produced by additional reactions that assess enzyme activity and carbon source utilization. During incubation at 35°C, the device takes panel measurements every 20 minutes. Usually, the final results are available within 16 hours. The Phoenix system is a crucial instrument for routine clinical microbiology as well as AMR surveillance because of its dependability, adaptability, and extensive diagnostic capabilities [27].

MicroScan WalkAway: More than 100 laboratories in India have adopted the automated MicroScan WalkAway (Beckman Coulter Diagnostics) system for microbial ID/AST [13]. The device can process 40 or 96 panels at once and is offered in the Plus and DxM versions. Three panel formats, conventional, fast, and specialty, are used to accomplish identification. With Gram-positive and Gram-negative panels that can recognize 51 and 136 species, respectively, including ESBL confirmation, conventional panels combine ID and AST. Rapid ID panels can identify a wide range of Gram-positive and Gram-negative organisms in 2.5 hours by using fluorogenic substrates to detect pre-formed enzymes. Fastidious pathogens, yeasts, and anaerobes can be quickly identified using specialty panels; results are available in about four hours. The versatility, speed, and comprehensive coverage of the MicroScan WalkAway system make it an essential diagnostic platform for clinical microbiology laboratories and AMR monitoring [27].

Matrix-assisted laser desorption ionization-time of flight mass spectrometry: In clinical microbiology, matrix-assisted laser desorption ionization-time of flight mass spectrometry (MALDI-TOF MS) has drawn interest as a cutting-edge method for detecting carbapenemase activity. By tracking antibiotic breakdown products, this method can identify β-lactam hydrolysis in addition to its well-established use for rapid microbial identification. While initial research has shown potential for identifying plasmid-associated carbapenemase genes, present applications are limited to single-gene detection. The creation of an extensive database that includes frequently circulating plasmids and their corresponding carbapenemase genes is crucial to facilitating wider clinical application. Despite these drawbacks, MALDI-TOF MS's rapid turnaround time, precision, and versatility make it a useful supplementary tool to traditional and molecular diagnostic techniques in the battle against carbapenem resistance. MALDI-TOF MS detects carbapenemase activity by identifying hydrolysis products of carbapenem antibiotics. It provides rapid results within one to two hours and has demonstrated high concordance with molecular methods; however, its implementation is limited by equipment costs and database requirements [28].

Automated broth microdilution systems: By standardizing endpoints and lowering variability when compared to manual approaches, automated broth microdilution platforms have revolutionized AST. The US Food and Drug Administration (FDA) has approved these systems, which come in semi-automated and fully automated configurations and usually provide results overnight or within 16 hours of incubation. Manufacturers simplify data processing and reporting by offering a variety of panel designs, capabilities, and specialized software. Reduced labor requirements, increased repeatability, improved data management, and rapid generation of clinically meaningful results are the main benefits of automated AST systems. Crucially, prompt dissemination of these findings to physicians and pharmacists is necessary to enhance patient care, support antimicrobial stewardship, and maximize the benefits of rapid diagnostics [29]. Comparative analysis of conventional and automated methods (Table 1).

**Table 1:** Comparative performance of phenotypic, molecular, and automated methods for carbapenemase detection Comparative analysis of conventional and automated methods for carbapenemase detection, highlighting their sensitivity, specificity, turnaround time, and suitability for use in clinical microbiology laboratories. MHT: modified Hodge test; MIC: minimum inhibitory concentration; MALDI-TOF MS: matrix-assisted laser desorption ionization-time of flight mass spectrometry; ESBL: extended-spectrum β-lactamase; CDT: combined disk test; DDST: double disk synergy test; WGS: whole genome sequencing; mCIM: modified carbapenem inactivation method; eCIM: ethylenediaminetetraacetic acid-carbapenem inactivation method; PCR: polymerase chain reaction; MDR: multidrug-resistant; KPC: *Klebsiell pneumoniae* carbapenemase; CLSI: Clinical and Laboratory Standards Institute; OXA; MBL: metallo-β-lactamase; ID: identification; FDA: Food and Drug Administration; AST: antimicrobial susceptibility testing

Method	Sensitivity (%)	Specificity (%)	Time	Suitability
Disk diffusion	80%-90%	80%-85%	18-24 h	Routine labs, resource-limited settings
MIC (broth/agar dilution, E-test)	95%-100%	95%-100%	16-24 h	Gold standard, quantitative resistance
MHT	70%-85%	60%-75%	18-24 h	Simple setup, KPC detection
CDT	90%-95%	90%-95%	18-24 h	Differentiates carbapenemase classes
DDST	90%-95%	90%-95%	18-24 h	ESBL detection in Enterobacteriaceae
Carbapenem inactivation method (CIM/mCIM)	95%-100%	95%-100%	24 h	Reliable, CLSI standardized
Carba NP test	95%-100%	95%-100%	2 h	Rapid phenotypic confirmation
Blue-Carba test	90%-100%	85%-95%	2 h	Cost-effective, limited OXA-48 detection
EDTA-based assays (mCIM/eCIM)	95%-100%	95%-100%	24 h	Differentiates MBL vs serine carbapenemases
Boronic acid assay	90%-95%	90%-95%	18-24 h	Class A carbapenemase detection
Conventional PCR	95%-100%	95%-100%	4-6 h	Reliable detection of known carbapenemase genes
Multiplex PCR	95%-100%	95%-100%	4-6 h	Detects multiple genes simultaneously
GeneXpert Carba-R assay	95%-100%	85%-95% (lower for OXA-48/181)	~52 min	Rapid cartridge-based detection
Check-MDR Carba assay	95%-100%	95%-100%	~5.5 h	Multiplex PCR for rectal/throat swabs
WGS	98%-100%	98%-100%	24-72 h	Comprehensive detection, epidemiology, outbreak tracking
Automated systems (VITEK 2, BD Phoenix, MicroScan)	95%-100%	95%-100%	4-18h	Hospital labs, surveillance
MALDI-TOF MS	95%-98%	95%-98%	1-2h	Advanced labs, rapid ID
Automated broth microdilution	95%-100%	95%-100%	≤16 h	FDA-approved, reproducible AST

Clinical applications and importance

Early and accurate detection of carbapenemase-producing organisms is vital for effective patient management, as it enables timely initiation of appropriate therapy and reduces the risk of treatment failure. In healthcare settings, such diagnostics play a vital role in infection prevention and control by limiting nosocomial transmission and protecting vulnerable patient populations. They also serve as a cornerstone of antimicrobial stewardship programs, guiding rational antibiotic use and minimizing unnecessary carbapenem exposure. Furthermore, hospital surveillance systems depend on reliable detection methods to monitor resistance trends, identify high-risk clones, and implement outbreak control measures. Collectively, these applications underscore the importance of carbapenemase diagnostics in improving patient outcomes, strengthening infection control strategies, and supporting public health initiatives.

Challenges in carbapenemase detection

Despite advances in diagnostic technologies, several challenges continue to hinder accurate carbapenemase detection in clinical microbiology. False-positive and false-negative results remain a significant limitation, particularly with phenotypic assays that may misinterpret intrinsic resistance mechanisms or yield inconsistent outcomes. The ongoing emergence of novel carbapenemase variants further complicates detection, as existing assays are not always capable of identifying newly evolving enzymes. In resource-limited settings, laboratories often face constraints in accessing advanced molecular platforms, leading to reliance on conventional methods with reduced sensitivity and specificity. A lack of standardized protocols across institutions contributes to variability in results and hampers data comparability. Additionally, infrastructure limitations and the high costs associated with automated systems and molecular assays restrict their widespread implementation. Collectively, these challenges underscore the urgent need for affordable, standardized, and adaptable diagnostic strategies to ensure reliable detection and effective infection control.

Emerging trends and future perspectives

**Figure 4:**
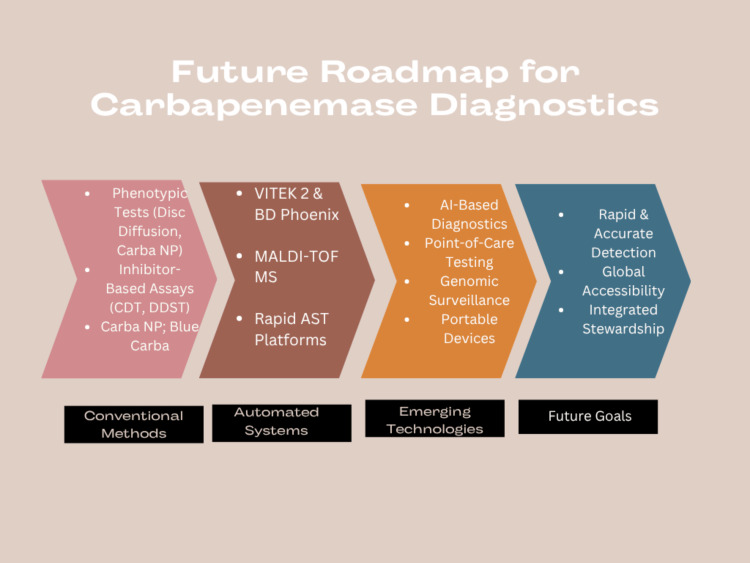
Future roadmap for carbapenemase diagnostics Future roadmap for carbapenemase diagnostics showing the evolution from conventional phenotypic methods and automated systems to emerging technologies such as AI-based diagnostics, point-of-care testing, and genomic surveillance, aiming for rapid, accurate, and globally accessible detection. CDT: combined disk test; DDST: double disk synergy test; MADI-TOF-MS: matrix-assisted laser desorption ionization-time of flight mass spectrometry; AST: antimicrobial susceptibility testing; AI: artificial intelligence Image credit: Created by authors using Canva (Canva Pty Ltd., Sydney, NSW, Australia).

The future of carbapenemase diagnostics is being shaped by several innovative approaches that aim to overcome current limitations. Artificial intelligence (AI) is increasingly applied to diagnostic platforms, offering enhanced accuracy through predictive algorithms and automated interpretation of complex datasets. Rapid point-of-care detection systems are being developed to provide bedside results, enabling immediate therapeutic decisions and improved patient outcomes. Genomic surveillance has become a powerful tool for tracking the spread of carbapenemase genes, supporting public health interventions and guiding infection control strategies. Integration of molecular assays with automated platforms is expected to decrease turnaround times, optimize workflows, and enhance diagnostic efficiency. Additionally, portable diagnostic devices are being recognized for their potential to deliver affordable, decentralized testing in resource-limited settings. Collectively, these emerging trends highlight a future roadmap toward faster, more precise, and accessible carbapenemase detection technologies that can strengthen both clinical practice and global AMR surveillance (Figure 4).

## Conclusions

One of the most urgent problems in contemporary clinical microbiology is the synthesis of carbapenemase by Gram-negative bacteria. In addition to directing successful antibiotic therapy, accurate detection is crucial for guiding infection control strategies and antimicrobial stewardship initiatives. Although they are frequently constrained by lower sensitivity, false positives, and longer turnaround times, conventional phenotypic tests, including disk diffusion, MIC testing, and inhibitor-based techniques, are nonetheless useful in resource-constrained settings because of their accessibility and affordability. Carbapenemase genes can be identified rapidly and with high specificity using molecular platforms such as multiplex PCR, GeneXpert Carba-R, and WGS, but their application is limited by cost and technical expertise. Hospital surveillance and outbreak investigations are supported by automated AST systems and cutting-edge technologies like MALDI-TOF MS, which further improve diagnostic speed and accuracy.

To guarantee accurate detection, an integrated diagnostic strategy that combines phenotypic screening with molecular confirmation is recommended, given the advantages and disadvantages of individual techniques. In order to promote comparability between laboratories and standardize interpretation, alignment with current CLSI and European Committee on Antimicrobial Susceptibility Testing (EUCAST) recommendations is essential. Ultimately, increasing the capacity to detect carbapenemases will improve patient outcomes, reduce the spread of resistant organisms, and aid international efforts to fight antibiotic resistance.
